# Development and validation of quality of life instruments for chronic diseases—Chronic gastritis version 2 (QLICD-CG V2.0)

**DOI:** 10.1371/journal.pone.0206280

**Published:** 2018-11-14

**Authors:** Peng Quan, Lei Yu, Zheng Yang, Pingguang Lei, Chonghua Wan, Ying Chen

**Affiliations:** 1 School of Humanities and Management, Research Center for Quality of Life and Applied Psychology, Guangdong Medical University, Dongguan, CHINA; 2 Huadu District, Guangzhou City People's Hospital, Guangzhou, CHINA; 3 School of Public Health, Guangdong Medical University, Dongguan, CHINA; 4 People’s Hospital of Songgang, Shenzhen, CHINA; 5 School of Public Health, Kunming Medical University, Kunming, CHINA; Public Library of Science, UNITED KINGDOM

## Abstract

Quality of life is an important outcome indicator to evaluate whether treatment is successful or not. Chronic gastritis leads to ongoing deterioration of subjectively perceived quality of life. There are several generic measures, but they are not developed particularly to assess chronic gastritis problems. The Quality of Life Instruments for Chronic Diseases—Chronic Gastritis (QLICD-CG V2.0) questionnaire is a 39-item, multi-dimensional, self-report instrument to assess chronic gastritis patients’ perception of their health related quality of life in four domains. The instrument was developed in China. The current study aimed to evaluate the psychometric properties of the QLICD-CG V2.0. 194 patients with chronic gastritis were enrolled from 4 hospitals in China. The QLICD-CG V2.0 was administered to patients by trained research assistants. In addition, their demographic characteristics were also recorded. The psychometric testing included construct validity, convergent validity, discriminant validity, test-retest, and responsiveness. The results showed good internal consistency and acceptable floor and ceiling effects (*Cronbach’s alpha* range from 0.80 to 0.93). CFA showed that the instrument structure has a reasonable fitness (*RMSE*A = 0.063, 95%CI = [0.057 0.079], *CFI* = 0.93, *GFI* = 0.95, *SRMR* = 0.028). The convergent validity was considered appropriate, with 38 of the 39 items correlated stronger with their assigned scale than a competing scale, except for GPS1. Known groups comparisons showed that the QLICD-CG V2.0 discriminated well between subgroups on the basis of gender, marriage status, and economy status, thus providing evidence of discriminative validity. Convergent validity testing revealed that the QLICD-CG V2.0 domain scores correlated significantly with SF-36 dimension scores, which ranged from 0.21 to 0.58. Test-retest coefficients were satisfactory. A majority of intraclass correlation coefficients were above 0.70, except the psychological domain (0.60) and the items of social support/security (0.61). Responsiveness was tested on 157 patients. Significant differences were found on all QLICD-CG V2.0 domains, between baseline responses and after a treatment, except for the items of appetite and sleep. Robust sensitivity to change was observed. The QLICD-CG V2.0 appears to be a valid and reliable instrument to measure QOL in chronic gastritis patients. Scores were reproducible.

## Introduction

Chronic gastritis has received an increasing attention within medical practice. It is a long-term inflammation of the gastric mucosa, which can significantly impair the quality of life (QOL) of the patients [1]. Although the progress in gastritis treatment has been remarkable, chronic gastritis still results in difficulties for patients’ everyday life, which leads to ongoing deterioration of QOL [2]. Accurate assessment of subjective feeling is critical to determining the efficacy of treatment.

Health related quality of life (HRQOL) reflects patients’ feelings and functioning and the impact of their health condition beyond simple symptom assessment [3]. Some generic HRQOL measures have been developed and widely used across a range of diseases, such as 36-item short form health survey (SF-36) [4], and WHO quality of life-BREF (WHOQOL-BREF) [5]. Most of the time, studies use generic measures to study quality of life of chronic gastritis patients. These generic measures place emphasis on overall life satisfaction, such as social functioning, and general health perceptions. They focus on general symptoms or function, e.g., pain. However, there are also some specific symptoms in chronic gastritis patients, such as bloating, heartburn, belching or nausea [6–8]. These symptoms cause deterioration in chronic gastritis patients. Generic measures fail to cover all these symptoms on QOL, and may not fully evaluate the entire range of QOL issues, which certain patients may experience. Although there are some specific disease-oriented questionnaires, such as Gastrointestinal Quality of Life Index (GLQI) [9] and EORTC QLQ-STO22 [10], several comments can be made about these questionnaires. GLQI and QLQ-STO22 are not disease-specific measures for chronic gastritis. Disease-specific questionnaires are more efficient than generic questionnaires [11]. Thus, a more specific HRQOL measure, developed particularly to assess chronic gastritis problems, would be useful in assessing HRQOL and to evaluate whether treatment is successful or not.

The purpose of the current study was to evaluate the psychometric properties of a new QOL instrument for chronic gastritis patients, the Quality of Life Instruments for Chronic Diseases—Chronic Gastritis (QLICD-CG V2.0).

## Materials and methods

The development of QLICD-CG V2.0 was conducted in a standardized manner, consisting of item development, pilot testing, and psychometric validation [12, 13]. The ethics committee of Guangdong medical university approved of this study. Written informed consents were obtained from all the participants prior to survey participation.

## Item development debriefing

This study focused on the specific module development across several domains for chronic gastritis patients. The QLICD-CG V2.0 is a self-report measure, with a total of 39 items covering a general module (QLICD-GM, including three domains: 9 items in physical domain, 11 items in psychological domain, and 8 items in social domain) and a specific module (11 items, including three disease-specific domain: epigastric pain, satiety, and psychological impact for chronic gastritis). Each item of the QLICD-CG V2.0 was scored on a 5-point Likert scale (possible score range: 1 to 5, ranging from 1 no problem, to 5 extreme problem). The maximum possible score range of the QLICD-CG V2.0 is 39–195 (28–140 is the maximum possible score range of the general module, 11–55 is the maximum possible score range of the specific module). In the QLICD-CG V2.0, higher scores represent better QOL. It takes about 20 minutes to complete the questionnaire.

Items were generated through a multi-step process: physician consensus panels, semi-structured patients’ interviews, and several revisions made in response to patients’ data and feedback. Firstly, a pool of 17 items was generated, which consisted of candidate items that reflected the construct concept of the specific module. Secondly, several semi-structured interviews focused on the impact of disease on QOL were conducted. The content derived from these interviews was examined in conjunction with review of relevant literatures and was consulted with 16 experts, including physicians and researchers in clinical and psychometric field. And third, a preliminary QLICD-CG V2.0 paper and pencil questionnaire was conducted with 30 patients. Semi-structured interviews were performed to assess patients’ interpretations of the questions. As shown in Table 1, data were gathered on demographic and clinical aspects of patients in preliminary test.

**Table 1:** Demographic characteristics of 30 patients in preliminary test.

	n		n
**Gender**		**Job**	
Male	15	Worker	13
Female	15	Peasant	8
		Teacher	2
**Age**		Official	3
<30	4	Freelance	4
30–39	11	Other	0
40–49	12		
50–59	3	**Marriage**	
≥60	0	Married	28
		Other	2
**Education**			
Primary school	8	**Economic status**	
Middle school	5	Poor	12
High school	11	Middle	13
2 year college	3	Good	5
Undergraduate and above	3	**Clinical subtype**	
**Medical insurance**		Superficial gastritis	7
Self-provided	5	Superficial gastritis with erosion	3
Urban worker medical insurance	12	Flattened erosive gastritis	10
Urban resident basic medical insurance	3	Bile reflux gastritis	4
Rural cooperative medical insurance	5	Complex gastritis	6
Commercial health insurance	5		

Fourth, after piloting, a formal QLICD-CG V2.0 of 11 items was produced. 174 chronic gastritis patients from four hospitals in China were enrolled in formal test. QLICD-CG V2.0 and QLICD-GM, together with a few questions on demographic and clinical features, were administered to patients by trained research assistants. All participants also answered the SF-36 at the same time.

The investigators described the study to the participants and obtained informed consent from those who agreed to participate and met the inclusion criteria. The inclusion criteria were: 18 years or older, capacity to consent [14]. Chronic gastritis was diagnosed primarily through endoscopy and gastric biopsy by the physician. The classification criteria of chronic gastritis was proposed by Chinese society of digestive endoscopy [15]. In order not to bias responses, the questionnaires were completed after a clinical examination to confirm that the patients were in a stable phase and before the medical procedures. Stable phase was defined as that patient reported no life events and no health changes.

### Study design and population

This study was conducted in Guangdong medical university affiliated hospital from July 2015 to May 2016. The outpatients with clinical symptoms of chronic gastritis were chosen as the subjects. A total of 194 subjects were requested to sign informed consent, to complete the paper and pencil questionnaires, and to examine the situation of gastric mucosa by the gastroscopy. All items were reported well understood. According to the results from the questionnaires, 20 patients were excluded for missing response to more than 50% of the total items, and 174 patients remained. Gastric mucosa divided 174 subjects into six clinical subtype groups such as superficial gastritis, superficial gastritis with erosion, flattened erosive gastritis, bile reflux gastritis, complex gastritis, missing. The missing data rate for each item varied from 3.14% to 5.68%. For missing responses, the mean was calculated by imputing the missing responses based on the mean of the non-missing items.

The inclusion criteria were as following: firstly, the patients were diagnosed as chronic gastritis. Secondly, there were no medical history of tumor.

### Clinical and demographic characteristics

As shown in Table 2, data were gathered on demographic and clinical aspects of patients in formal test. The types of gastritis (superficial gastritis, atrophic gastritis, gastric atrophy, non-erosive gastritis and non-specific gastritis) were recorded with biopsy examination and gastroscopy.

**Table 2:** Demographic characteristics of 174 patients in formal test.

	n	Percentage		n	Percentage
**Gender**			**Job**		
Male	79	45.40%	Worker	28	16.09%
Female	94	54.02%	Peasant	61	35.06%
Missing	1	0.57%	Teacher	10	5.75%
**Age**			Official	11	6.32%
<30	8	4.60%	Freelance	18	10.34%
30–39	24	13.79%	Other	45	25.86%
40–49	28	16.09%	Missing	1	0.57%
50–59	60	34.48%	**Marriage**		
≥60	52	29.89%	Married	157	90.23%
Missing	2	1.15%	Other	16	9.20%
**Education**			Missing	1	0.57%
Primary school	51	29.31%	**Economic status**		
Middle school	61	35.06%	Poor	52	29.89%
High school	40	22.99%	Middle	109	62.64%
2 year college	12	6.90%	Good	13	7.47%
Undergraduate and above	10	5.75%	**Clinical subtype**		
**Medical insurance**			Superficial gastritis	57	32.76%
Self-provided	116	66.67%	Superficial gastritis with erosion	33	18.97%
Urban worker medical insurance	6	3.45%	Flattened erosive gastritis	72	41.38%
Urban resident basic medical insurance	38	21.84%	Bile reflux gastritis	4	2.30%
Rural cooperative medical insurance	5	2.87%	Complex gastritis	1	0.57%
Commercial health insurance	9	5.17%	Missing	7	4.02%

### Statistical analysis

Data processing and statistical analyses were performed using SPSS 18 and Mplus 7. Quantitative variables were expressed as means ± standard deviations. The significance level was set at *p*< 0.05. Reliability measures were of two types: 1. Internal consistency reliability was assessed using *Cronbach’s alpha* coefficient calculated for each scale (a value > = 0.70 supported internal consistency reliability) [16]; 2. Test-retest reliability was assessed using *ICC* (intraclass correlation coefficient). The QLICD-CG V2.0 was administered twice to 101 patients whose clinical conditions were stable (defined by patient without reporting life events and health changes), separated by a one-week interval, to quantify reproducibility of scores. Responsiveness was tested using the standardized effect size on 157 patients who experienced an antibacterial and antiulcer treatment through which the patients’ health statuses changed. The QLICD-CG V2.0 was administered for a second time to these 157 patients within 6 months of the baseline visit to determine whether the instrument was sensitive to these changes in QOL.

Convergent and discriminant validity were examined by comparing the item-dimension correlation. Convergent validity was assessed by correlating each item with the scale it was hypothesized to belong to (a correlation *r* > = 0.4 supported item internal consistency). And discriminant validity was supported whenever a correlation between an item and its hypothesized scale was higher than its correlation with the other components. Floor and ceiling effects were assessed by the homogeneous response distribution of scores. A confirmatory factor analysis (CFA) was performed using the structural equation modeling. The following indexes were required: the Root Mean Square Error of Approximation (*RMSEA*) is acceptable if <0.08, the General Fit index (*GFI*) and Comparative Fit Index (*CFI*) are acceptable if >0.90, and the Standardized Root Mean Square Residual (*SRMR*) is acceptable near 0 [17, 18]. The construct validity was assessed using Pearson’s correlation coefficients of the domain scores of the QLICD-CG V2.0 with the SF-36. The discriminant validity was measured by the associations between the QLICD-CG V2.0 domain scores and demographic features and clinical characteristics. Student’s *t* tests and one-way ANOVA were used to compare the mean domain scores of the QLICD-CG V2.0 across different patient groups.

## Results and discussion

### Score distribution

**Fig 1:**
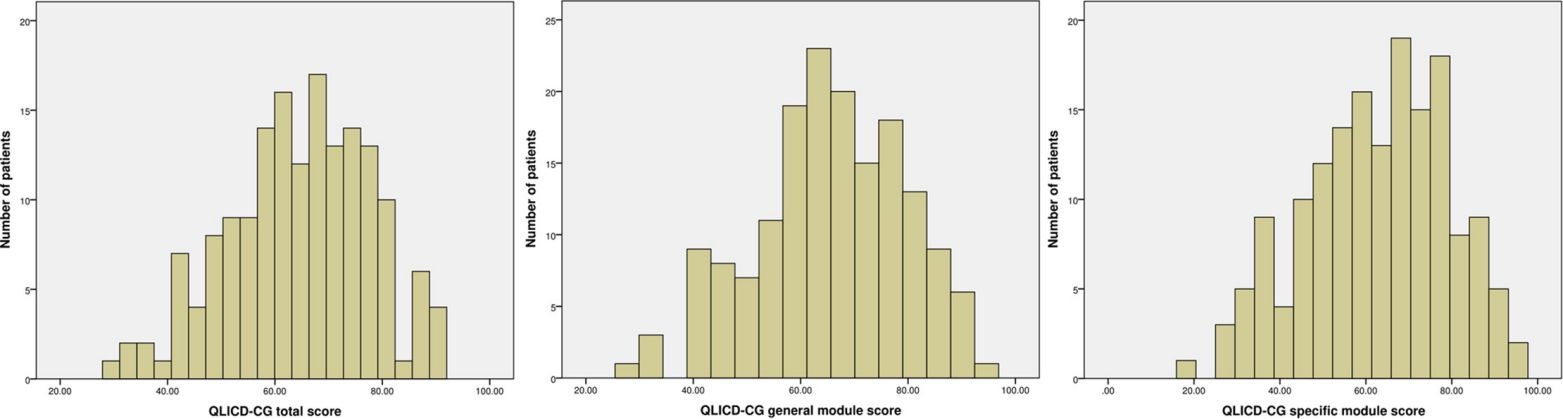
Histogram of QLICD-CG V2.0 total score, general module score, and specific module score.

The QLICD-CG V2.0 total mean score was 127.29±27.20, with a range 59.25–183.85 The general module mean score was 65.56±13.72, with a range 27.68–92.86. The specific module mean score was 61.73±16.64, with a range 31.57–90.99. As shown in Fig 1, the total score and module scores showed no floor or ceiling effect as none of patients obtained the minimum or the maximum score. (Fig 1)

### Construct validity

As shown in Table 3, *Cronbach a* for the QLICD-CG V2.0 domains ranged from 0.80 to 0.93, which showed good internal consistency [19]. The QLICD-CG V2.0 showed high internal consistency as demonstrated by the *Cronbach’s alpha* coefficients (>.80) on each domain. CFA also showed a reasonable fitness (*RMSE*A = 0.063, 95%CI = [0.057 0.079], *CFI* = 0.93, *GFI* = 0.95, *SRMR* = 0.028).

**Table 3:** General description, and internal consistency of the QLICD-CG V2.0.

	Items number	Cronbach's alpha
**Physical domain**	9	.80
**Psychological domain**	11	.88
**Social domain**	8	.82
**General module**	28	.91
**Specific module**	11	.85
**Total score**	39	.93

Construct validity was investigated via the known groups comparisons for discriminative validity. Table 4 shows Pearson correlation coefficients between items and the QLICD-CG V2.0 domains. 38 out of 39 items had the largest correlation coefficient for each item with their assigned domains, except for GPS1, which correlated *r* = 0.60 on physical domain, and *r* = 0.50 on psychological domain (difference = 0.10). We still chose to include GPS1 in the psychological domain which seemed more reasonable, even if the loading was lower in the psychological domain than the physical domain. Normally, a minimum dimension loading of 0.50 is recommended [20]. However, four items demonstrated low loadings on their assigned dimension (range 0.34–0.50; GPH3-Do you feel treatment cause sexual problem; GPS1-Can you focus on what you are doing, GPS3-Do you think life is fun, CG11- Do you feel annoyed for the restricted diet due to illness). Except for GPS1, three items (GPH3, GPS3, CG11) did not overlap others (loadings of ≥0.40 on more than one factor). These four items were discussed, and believed to be important to chronic gastritis patients. Therefore, we reserved these items in the QLICD-CG V2.0.

**Table 4:** Item-domain correlations on the QLICD-CG V2.0.

	Physical domain	Psychological domain	Social domain	Specific module
**GPH1**	0.53**	0.17*	0.32**	0.32**
**GPH2**	0.60**	0.36**	0.18*	0.32**
**GPH3**	0.42**	0.15	0.15*	0.08
**GPH4**	0.51**	0.23**	0.23**	0.20**
**GPH10**	0.63**	0.51**	0.19*	0.47**
**GPH6**	0.68**	0.29**	0.47**	0.32**
**GPH7**	0.77**	0.36**	0.49**	0.33**
**GPH8**	0.71**	0.30**	0.39**	0.35**
**GPH9**	0.71**	0.44**	0.32**	0.44**
**GPS1**	0.60**	0.50**	0.31**	0.34**
**GPS2**	0.40**	0.58**	0.05	0.41**
**GPS3**	0.25**	0.34**	0.29**	0.15
**GPS4**	0.20**	0.69**	0.20**	0.43**
**GPS5**	0.26**	0.71**	0.54**	0.34**
**GPS6**	0.29**	0.75**	0.31**	0.37**
**GPS7**	0.43**	0.88**	0.46**	0.48**
**GPS8**	0.40**	0.81**	0.45**	0.44**
**GPS9**	0.30**	0.74**	0.37**	0.43**
**GPS10**	0.47**	0.60**	0.51**	0.30**
**GPS11**	0.29**	0.68**	0.30**	0.52**
**GSO1**	0.49**	0.39**	0.70**	0.23**
**GSO2**	0.25**	0.30**	0.66**	0.14
**GSO3**	0.22**	0.32**	0.64**	0.15
**GSO4**	0.45**	0.27**	0.74**	0.23**
**GSO5**	0.34**	0.26**	0.68**	0.20**
**GSO6**	0.24**	0.44**	0.58**	0.29**
**GSO7**	0.27**	0.42**	0.67**	0.28**
**GSO8**	0.48**	0.34**	0.72**	0.16*
**CG1**	0.32**	0.53**	0.29**	0.70**
**CG2**	0.36**	0.49**	0.31**	0.68**
**CG3**	0.56**	0.39**	0.40**	0.67**
**CG4**	0.54**	0.44**	0.28**	0.75**
**CG5**	0.35**	0.38**	0.09	0.70**
**CG6**	0.32**	0.36**	0.01	0.64**
**CG7**	0.29**	0.19*	0.27**	0.55**
**CG8**	0.30**	0.41**	0.10	0.66**
**CG9**	0.07	0.29**	0.10	0.53**
**CG10**	0.26**	0.26**	0.23**	0.57**
**CG11**	0.25**	0.26**	0.14	0.43**

GPH physical domain, GPS psychological domain, GSO social domain, CG specific module for chronic gastritis

* *p*<0.05 level

**p<0.01.

As shown in Table 5, the discriminant validity of QLICD-CG V2.0 was assessed using the domain scores of the QLICD-CG V2.0 across patient groups with different demographic and clinical characteristics. Student’s *t* tests and one-way ANOVA were used to compare these mean domain scores. Females reported significantly lower scores in the psychological domain. Single patients reported significantly lower scores in the psychological domain, general module, and total score. Patients with less income reported significantly lower scores in all domains, except physical domain.

**Table 5:** Comparisons of QLICD-CG V2.0 domain scores with respect to patients’ demographic and clinical features.

	Physical domain	Psychological domain	Social domain	General module	Specific module	Total
**Gender**						
**Male**	61.38±16.34	69.24±16.12	70.21±15.00	66.99±13.15	62.33±15.97	65.67±12.41
**Female**	61.30±14.72	62.28±19.57	70.79±16.07	64.40±14.22	61.31±17.33	63.53±13.91
*p*	0.97	0.02*	0.82	0.23	0.71	0.32
**Marriage status**						
**Married**	62.03±14.99	67.00±17.86	71.01±15.64	66.55±13.51	62.52±16.20	65.41±12.91
**Other**	54.63±18.02	49.24±15.26	66.25±13.89	55.83±12.24	53.94±19.48	55.30±13.10
*p*	0.08	0.00*	0.26	0.00*	0.06	0.00*
**Education**						
**Below college**	60.92±15.05	64.38±17.80	70.10±15.39	64.90±13.20	60.93±16.18	63.78±12.57
**College or higher**	64.44±17.67	72.39±20.92	73.91±16.39	70.27±16.67	67.39±19.20	69.46±16.72
*p*	0.34	0.07	0.31	0.10	0.11	0.07
**Economy status**						
**Poor**	59.75±17.23	59.42±17.87	65.24±17.36	61.19±14.47	57.65±18.88	60.19±14.09
**Good**	62.04±14.54	67.92±18.00	72.86±14.12	67.44±13.01	63.48±15.35	66.32±12.43
*p*	0.39	0.01*	0.00*	0.01*	0.04*	0.01*
**Medical insurance**						
**Self-pay**	60.49±23.06	65.40±18.21	77.43±12.58	67.26±15.62	64.14±11.41	66.38±13.23
**Insurance**	61.40±14.92	65.36±18.40	70.17±15.61	65.46±13.66	61.59±16.92	64.37±13.25
*p*	0.86	0.99	0.17	0.70	0.66	0.66
**Clinical subtype**						
**Type1**	61.7	64.12±16.56	72.18±13.26	65.64±11.64	61.10±16.88	64.36±11.78
**Type2**	61.11	71.14±19.30	74.24±14.72	68.80±14.30	63.02±14.50	67.17±13.43
**Type3**	61.53	63.70±19.43	66.84±17.53	63.90±15.28	61.33±17.22	63.17±14.51
**Type4**	53.7	53.79±26.63	66.67±15.42	57.44±19.16	64.39±27.11	59.40±20.21
**Type5**	72.22	75	78.13	75	65.91	72.44
*p*	0.88	0.26	0.17	0.39	0.98	0.61

Clinical subtype: type1 superficial gastritis, type2 superficial gastritis with erosion, type3 flattened erosive gastritis, type4 bile reflux gastritis, type5 complex gastritis

* *p* < .05.

### Convergent validity

Table 6 shows Pearson’s correlation coefficients of the domain scores of the QLICD-CG V2.0 with the SF-36. Results indicated positive correlations. If there were higher correlations between corresponding than non-corresponding domains, the convergent validity was good.

**Table 6:** Pearson’s correlation coefficients of the QLICD-CG V2.0 with the SF-36.

	Physical functioning (SF-36)	Physical role functioning (SF-36)	Bodily pain (SF-36)	General health (SF-36)	Vitality (SF-36)	Social role functioning (SF-36)	Emotional role functioning (SF-36)	Mental Health (SF-36)
**Physical domain (QLICD-CG V2.0)**	.54**	.40**	.40**	.47**	.39**	.41**	.29**	.33**
**Psychological domain (QLICD-CG V2.0)**	.32**	.26**	.37**	.61**	.58**	.35**	.39**	.55**
**Social domain (QLICD-CG V2.0)**	.22**	.30**	.43**	.26**	.19*	.39**	.22**	.30**
**Specific module (QLICD-CG V2.0)**	.32**	.35**	.48**	.41**	.36**	.31**	.21**	.31**

** *p* < .01

### Test-retest reliability and responsiveness

Test-retest reliability was evaluated with *ICC*. The QLICD-CG V2.0 was administered to 101 patients at baseline and one week after the baseline visit. As shown in Table 7, a majority of *ICCs* were above .70, except the psychological domain (.60) and the item of social support/security (.61). Responsiveness was assessed using the standardized effect size on 157 patients who experienced a treatment within 6 months of the baseline visit. Standardized effect sizes were calculated by use of the formula: Effect size = (baseline QOL score—QOL score after a treatment) / Standard deviation of change QOL scores. The mean duration of time between baseline and post-treatment assessments was 130.82 ± 28.65 days. Significant differences were found between baseline responses and after a treatment, except for the items of appetite and sleep.

**Table 7:** Test-retest reliability and responsiveness of the QLICD-CG V2.0.

	*ICC*	Standardized effect size	Before treatment		After treatment	*p*
**Physical domain**	.88	.83	61.66±15.41		69.27±14.78	.00
Independence	.75	.88	51.59±14.60		61.74±15.22	.00
Appetite and sleep	.84	.71	80.47±22.97		81.95±1.80	.27
Physical symptoms	.74	.82	53.58±23.22		65.29±21.95	.00
**Psychological domain**	.60	.78	65.84±18.02		73.74±17.56	.00
Cognition	.83	.82	69.43±20.14		74.52±19.14	.00
Emotion	.79	.86	64.65±20.39		73.32±20.50	.00
Will and personality	.77	.82	66.40±20.50		74.44±17.98	.00
**Social domain**	.75	.86	70.72±15.56		76.05±13.78	.00
Social support/security	.61	.73	75.96±15.65		81.53±14.54	.00
Social effects	.76	.75	68.42±17.95		73.78±17.03	.00
Sexual function	.73	.62	66.32±20.54		71.26±18.04	.00
**General module**	.80	.78	65.89±13.69		72.96±13.43	.00
**Specific module**	.76	.92	61.97±16.45		71.55±15.44	.00
Epigastric pain	.77	.76	62.94±20.10		75.20±17.75	.00
Satiety	.71	.63	64.09±19.91		74.44±18.97	.00
Psychological impact for chronic gastritis	.77	.90	57.86±20.83		62.85±19.30	.00
**Total score**	.79	.85	64.78±13.12		72.57±12.94	.00

## Discussion

Chronic gastritis may damage stomach for years, affecting patients’ health and subjective perceived quality of life. There are particular challenges in chronic gastritis patients’ life, including the physical impairment, but also change in psychological and social domain [21]. These challenges are reflected in the QLICD-CG V2.0, but are not captured in other generic instruments [9, 10]. The impact of disease-specific impairment on chronic gastritis patients has received little attention in research and clinical practice, and the QLICD-CG V2.0 provides a tool to address this gap.

This validation study of the QLICD-CG V2.0 showed a 4-domain structure, in a population of Chinese patients with current chronic gastritis. The QLICD-CG V2.0 showed good construct validity, convergent validity, discriminant validity, test-retest reliability, and responsiveness. The convergent validity was supported through positive correlations between the domain score of the QLICD-CG V2.0 and the SF-36. Results also showed that the QLICD-CG V2.0 is suitable for longitudinal studies to detect a meaningful change in chronic gastritis patients. As a disease-specific instrument that focuses on particular symptoms, the QLICD-CG V2.0 is sensitive enough to detect any small changes in QOL. This instrument could be used to monitor response to treatment, which will be applicable to research studies as well as to clinical practice.

The QLICD-CG V2.0 has at least two interesting specificities. First, disease-specific module is of particular interest. There are particular challenges in chronic gastritis patients, including the physical, psychological, social, and disease-specific impairment. These challenges are reflected in the QLICD-CG V2.0, but has been unexplored by other generic QOL instruments. The impact of disease-specific impairment on chronic gastritis patients has received little attention in research and clinical practice, and QLICD-CG V2.0 provides a tool to address this gap. The development process of QLICD-CG V2.0 could contribute to the acknowledgement of the importance of the patient perspective in the treatment and outcome assessment. Thus, QLICD-CG V2.0 can add important value to patient recovery.

Second, the QLICD-CG V2.0 was validated on a broadly representative group of chronic gastritis patients, which included superficial gastritis, atrophic gastritis, gastric atrophy, non-erosive gastritis and non-specific gastritis. This captures all aspects of chronic gastritis patients’ QOL. Furthermore, to complete the QLICD-CG V2.0 only needs about 20 minutes, making the QLICD-CG V2.0 compatible for clinical practice.

There are some limitations of the present study to be considered. First, we did not test longitudinal responsiveness. It is important to investigate whether the QLICD-CG V2.0 can detect changes in the long run. Second, the sample may be not representative enough. Our study enrolled patients in hospital, the findings might not generalize to those patients who don’t come to hospital. Moreover, we failed to discriminate patients with different clinical subtype. It might be due to the small sample size of our study. There are 4 patients with bile reflux gastritis, and only one patient with complex gastritis. Third, there are different known causes of chronic gastritis. A specific cause is difficult to be identified. It is hard to explain the differences between different demographic groups, and thus the generalizability of research findings is uncertain. Further validation of the QLICD-CG V2.0 is needed. A larger sample can yield more accurate results. Future research should explore possible explanations for the differences between different demographic groups.

## Conclusion

The QLICD-CG V2.0 is an instrument to assess QOL among patients with chronic gastritis, which presents good psychometric properties. To date, QLICD-CG V2.0 is the only QOL instrument specific to chronic gastritis patients. The QLICD-CG V2.0 can be used in the context of research studies as well as to clinical practice. However, it should be noted that further examination and confirmation of its psychometric properties should be performed in other independent samples.

## Supporting information

S1 Appendix QLICD-CG V2.0 dataset. (SAV)
